# 2637. Activity of Respiratory Viruses in Georgia 2020-2022

**DOI:** 10.1093/ofid/ofad500.2249

**Published:** 2023-11-27

**Authors:** Ann machablishvili, Olgha Tarkhan-Mouravi, Khatuna Zakhashvili, Irina Kalandadze, Irakli Karseladze, Mari Gavashelidze, Paata Imnadze

**Affiliations:** National Center for Disease Control and Public Health, Tbilisi, Georgia; National Center for Disease Control and Public Health, Tbilisi, Georgia; National Center for Disease Control and Public Health, Tbilisi, Georgia; National Center for Disease Control and Public Health, Tbilisi, Georgia; National Center for Disease Control and Public Health, Tbilisi, Georgia; National Center for Disease Control and Public Health, Tbilisi, Georgia; National Center for Disease Control and Public Health, Tbilisi, Georgia

## Abstract

**Background:**

The emergence of SARS-CoV-2 in early 2020 with subsequent implementation of public health and social measures influenced the epidemiology of respiratory viruses worldwide. This study describes circulation of respiratory viruses during two winter seasons and inter-seasonal periods in Georgia.

**Methods:**

We aggregated data obtained from Influenza like illness (ILI) and Severe Acute Respiratory Infection (SARI) cases during 2020/21 and 2021/22 (weeks 40-20) and inter-seasonal (weeks 21-39) periods in Georgia. All specimens from patients were screened on 22 respiratory viruses by real time RT-PCR.

**Results:**

During 2020/21 season 50% (868/1729) specimens tested positive on at least one virus. No influenza virus and few RSV detections were observed in Georgia. Mostly identified virus was Rhino (44.8%) circulating almost all season; followed by Coronavirus OC43 (23.7%), primarily seen in weeks 12-18 and SARS-Cov-2 (15%) with highest detection rates during weeks 48-52. In 2021 inter-season 54% (825/1513) of specimens were positive. A gradual increase in RSV detections was observed in summer. Most seen viruses were Rhino (30%), HMPV (22%) and Parainfluenza virus 3 (21%) circulating mainly in June and July. During 2021/22 season 44% (2012/4545) of specimens screened positive, out of which RSV (25.7%) with earlier circulation in weeks 40-2 and Rhino (25.7%) with most detections in the beginning and at the end of the season. Unusually influenza A/H3 (17%) peaked in late spring (weeks 12-18). SARS-Cov-2, Parainfluenza viruses 3 and 4, Adenoviruses, Human bocavirus, enteroviruses were actively co-circulating too. In 2022 inter-season 54% (1320/2456) of samples tested positive. Influenza A/H3 circulation continued until July. SARS-Cov-2 (27.7%), Rhino (22%), HMPV (14.5%), Parainfluenza virus 3 (12.7%), RSV (10.8%) were mostly seen viruses predominantly in June.

**Conclusion:**

Wider range respiratory viruses with higher positivity rates were detected among ILI and SARI cases in 2021/22 season compared to 2020/21 presumably due to mitigation of public health and social measures. Low detection rates of SARS-CoV-2 at sentinel sites could be explained by referral of COVID-19 patients to dedicated clinics. RSV and Influenza circulations were unusual in summer months in Georgia.

**Disclosures:**

**All Authors**: No reported disclosures

